# Multitechnique Physicochemical Characterization of Horse Hoof Trimmings as Sustainable and Untapped Keratin Source

**DOI:** 10.1002/open.70300

**Published:** 2026-09-21

**Authors:** Esther Trigueros, Lisa Rita Magnaghi, Lorenzo De Vita, Lavinia Rita Doveri, Michela Sturini, Chiara Milanese, Raffaela Biesuz

**Affiliations:** ^1^ Dipartimento di Chimica Università degli Studi di Pavia Pavia Italy; ^2^ Department of Chemical Engineering and Environmental Technology School of Industrial Engineering University of Valladolid Valladolid Spain; ^3^ Institute of Sustainable Processes University of Valladolid Valladolid Spain; ^4^ INSTM, Unità di Ricerca di Pavia Firenze Italy

**Keywords:** horse hoof trimmings, keratinous wastes, physicochemical characterization, principal components analysis, thermal properties

## Abstract

In the keratin‐rich biomass scenario, horse hoof trimmings play a marginal role due to the limited volume and the scarce background on material features, despite added values such as cruelty‐free, localized and routine production and traceability. This work presents a multitechnique characterization of horse hooves, including physicochemical, structural, thermal, and compositional analysis, providing a solid background knowledge of material features, mandatory for upcycling strategy definition. FT‐IR, Raman spectroscopy, X‐ray powder diffraction, SEM‐EDX, thermogravimetric analysis, and elemental characterization are conducted on powdered samples obtained using various approaches. The material exhibited structural features typical of hard α‐keratin, with semicrystalline organization and high thermal stability. A relatively high higher heating value (21.9 MJ kg^−1^) highlights its potential for energy recovery routes, while structural robustness suggests suitability for material‐oriented valorization pathways. Lastly, the issue of metal contamination, attributable to shoeing practices, is systematically evaluated and correlated with shoeing habits, sampling region, and milling. Overall, this study positions horse hoof waste as a promising alternative keratin‐based biomass and provides the necessary physicochemical framework to support its integration into circular bioeconomy strategies.

## Graphical abstract

Horse hoof trimmings, cruelty‐free and traceable keratin‐rich waste, were characterized for particle size, morphology, thermal stability, composition and metal contamination. Results show a high higher‐heating value and promising upcycling paths for energy and materials, but variable metal contamination (shoeing‐ and milling‐dependent) requires optimized pretreatment.

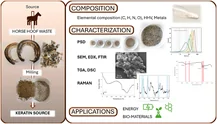

**Abbreviations**
AM: after milling
ATR: attenuated total reflectance
BM: before milling
CG: coffee grinder
db: dry basis
DSC: differential scanning calorimetry
DTG: derivative thermogravimetry
EDX: energy dispersive spectroscopy/X‐ray
FT‐IR: Fourier transform infrared spectroscopy
GHG: greenhouse gas
HH: horse hooves
HHV: higher heating value
ICP‐OES: inductively coupled plasma optical emission spectrometry
PCA: principal component analysis
PSD: particle size distribution
RT: room temperature
SEM: scanning electron microscopy
TGA: thermogravimetric analysis
WF: wood file
XRPD: X‐ray powder diffraction.

## 1 Introduction

The challenge of keratin‐based wastes can no longer be deferrable, mainly due to the ever‐increasing waste volumes derived from breeding and other livestock‐related industries (wool and leather production mainly) [1, 2]: keratin‐rich wastes from meat production globally increased from 10 million tonnes per year in 2012 to 11.82 after ten years, and a trend reversal is not expected so far since a global increase in meat protein consumption of 14% is expected in the next decade [1, 3, 4].

More than 60% of keratinous wastes generated annually are disposed of as refuse in landfills, dumpsites, and incinerators without adequate recycling, resulting in dramatic environmental drawbacks [2, 5]. As a matter of fact, each of these strategies poses peculiar challenges, ranging from landscape deterioration, development of malodor, pathogens, pests, and vermin, eutrophication, and algal bloom, in the case of disposal, or GHG release and pollution, in the case of incineration [2, 4].

Such a serious panorama strongly required efficient and scalable approaches to prevent keratin‐rich waste accumulation and promote their valorization through alternative management routes inspired by circular economy principles [2]. Livestock keratin valorization and application are generally harder than other kinds of wastes, such as plant‐derived polysaccharides [1, 6]: their unique structure and safety concerns about animal byproducts, which strongly increased after the mad cow and avian flu pandemics [2], are undoubtedly the main critical aspects. For this reason, keratin wastes are generally relegated to low‐value applications in large‐scale environments, such as biofertilizers and animal feed [7, 8]; the few examples of high‐value ones are limited to lab scale, mainly in the field of cosmetic products, biomedical devices, packaging materials, bio and superabsorbents, and geotextiles, as deeply discussed in recent reviews [1, 3, 5, 6, 7, 9, 10, 11].

Taking into account the different keratin sources, the annual global generation of keratinous materials stands at about 1–2 Mt of sheep wool, 8–10 Mt of poultry feathers, 4–6 Mt of human hair, and 1.2 Mt of horns and hooves [2]. In this context, poultry feathers have been mostly targeted to identify upcycling strategies for this type of waste, mainly pushed by the evident discrepancy of volumes and the easy collection from slaughtering plants [12]. For this reason, thousands of applications have been proposed for feathers, mainly focusing on chicken ones [8, 12, 13], followed by sheep wool, that share with feathers the localized production and huge volume, while the literature is definitively lacking in the case of other sources [12, 14, 15, 16].

In this panorama, we focused our attention on horse hooves (HHs) waste, derived from shoeing and trimming operations, being a sustainable, traceable, and cruelty‐free keratin source [17]. Such a waste material possess several pros that needs to be properly highlighted: first, trimming or shoeing must be around monthly conducted for horses’ health and well‐being; thus, these wastes are daily generated without implying any cruelty towards animals but even aiming at their well‐being [17]. For this reason, throughout the entire manuscript, we use the term “cruelty‐free” to clearly distinguish it from keratinous waste that is collected from animal corpses during slaughtering activities, with horse trimming obviously not lethal and mandatory to ensure horse well‐being during its daily life in stables and to prevent serious pathologies. Establishing whether daily trimming procedures are completely cruelty‐free, i.e., do not absolutely provoke any kind of suffering to the horses, falls out of the scope of this paper.

Second, hooves health, quality, and robustness are mandatory to guarantee the horses performances, resulting in peculiar attention to basal nutrition, dietary supplements, balanced work out, and proper housing approach and, eventually, in a higher quality and safety waste material rather than hooves from cattle slaughtering [17].

Third, a complete and detailed traceability can be guaranteed, being the shoeing or trimming activity singularly performed on each horse by specialized farriers and thus allowing one to exactly know not only the geographical provenience of the waste but also any other required detail, referred to the animal of origin (diet, habits, sport activity, sex, age, and shoeing procedure) [17]. This latter aspect plays a relevant role, especially in the first steps of the investigation, in which waste variability might be overwhelming, and having a bigger amount of metadata about the materials can help in dealing and controlling variability.

To conclude, a negative point must be mentioned about these alternative keratin sources: no detailed and clear information has been collected about this waste stream volume: A tentative estimation of a yearly production worldwide around 106,000 tons has been recently proposed [17], undoubtedly negligible if compared to the yearly production of keratinous animal byproducts, but nevertheless sufficient to justify the investigation on possible upcycling strategies.

Lastly, upcycling strategies for keratin‐based wastes can be generally divided into two main groups. The most popular ones involve the extraction of keratin from wastes according to various methodologies, deeply described elsewhere and here omitted for brevity’s sake [1, 2, 3, 5, 7, 11, 12, 13, 15, 18]; a less common but nevertheless interesting approach relies on the conversion of keratin wastes into powders to improve material properties, in the case of textiles, coatings, polymeric composites, materials for bioremediation, drug delivery, and tissue engineering [19, 20, 21, 22]. In both cases, a preliminary characterization of the waste of interest is mandatory to develop the best upcycling strategy for it, as smartly suggested both by S. Alvarez et al. [14] and by T. Tesfaye et al. [23].

Having this assumption clear in our mind, the main goal of the present investigation is the characterization of HH waste, in terms of physicochemical, chemical, and thermal properties but also humidity and water sorption and metal contamination, often hinted at as an unsolvable issue, especially in shod horses [24, 25, 26]. Additionally, we try to compare, as much as possible, the obtained results with other keratin‐rich waste, already characterized [14, 16, 19, 20, 23, 27], to better insert this untapped keratin source in the existing panorama.

## 2 Experimental Section

### 2.1 Sample Collection and Preparation

#### 2.1.1 Sampling Protocols

A pool of horses intended for equestrian activity and housed in indoor stables is selected: the sampling campaign includes horses characterized by different sex, age, breeds, dietary, shoeing, housing, and workout habits; horses presenting any type of hoof pathology have been excluded from the panel test. Since such a variability is unavoidable when collecting this type of waste, we decided to include it directly in the preliminary investigation. HH samples are collected from local stables (Lombardia, Italy) the same day of trimming procedures, thanks to the collaboration of local farriers, and immediately taken to the laboratory.

#### 2.1.2 Sample Cleaning and Storage

HH samples underwent an initial cleaning step to remove large particles and dirt by means of a brush. When required by subsequent processing, the hooves were chopped into smaller, manageable particles (around 200 mg each). The whole hooves and the resulting pieces were then oven‐dried at 70 °C for 24 h to preserve sample quality and prevent deterioration or bacterial growth. The moisture content is determined from the weight difference before and after drying. The dried samples were then stored in plastic bags at −18 °C until further use or analysis. The residual moisture is determined by heating the dried HH samples in an oven at 105 °C until a constant weight is achieved. This value is used to express the chemical composition on a dry basis (db). Before usage, HH samples undergo two further cleaning steps in distilled water and acetone 1 M to remove residual oil and grease, commonly used for aesthetic and health reasons, each one in an ultrasonic bath for 1 h at RT, followed by overnight drying at 70 °C.

#### 2.1.3 Milling Procedures

Milling is mandatory for both the characterization and the subsequent applications of the HH waste material, but it represents a critical task due to the high resistance and hardness of the material. In this investigation, we compared the following two strategies in terms of powder particle size:

1. Manual milling was carried out by wood file (WF) using the as‐received HH.
2. Pulverization by coffee grinder (CG) of the previously prepared small pieces repeating cycles of 1 min ON and 5 min OFF
3. Automated milling by cutting mills (CM) by FRITSCH Universal Cutting Mill Pulverisette 19 and 14 in collaboration with Emme 3 Srl (Via Bergamo 8, Lainate, MI 20045)

#### 2.1.4 Particle Size Distribution (PSD) Measurements

To determine the PSD of the powders obtained by the manual strategies, the samples are first fractionated using a series of bottom sieves with aperture sizes of 90, 106, 125, 149, and 212 µm. All the resulting fractions are weighted to determine the mass composition of the mixtures and then further characterized by laser diffraction with a LT3600Plus particle size analyzer (Linkoptik Instruments, Zhuhai, China) equipped with a 20 mW, 638 nm laser and a Venturi dry sample dispersion unit. For the latter automated approach, sieves of different aperture sizes are directly implemented in the millers, and 3 of them are tested: 200, 500, and 750 µm.

### 2.2 Physicochemical Characterizations

#### 2.2.1 SEM and EDX Analysis

Scanning electron microscopy (SEM) is performed on the manually milled HH samples sputtered with a thin gold film by a Zeiss EVO MA10 (Carl Zeiss, Oberkochen, Germany) instrument. The images are acquired at high voltage (20 kV) at working distance of 8.5 and 9 mm, under high vacuum, at room temperature, and at different magnifications, on 5 different regions of each sample surface.

#### 2.2.2 X‐Ray Powder Diffraction Analysis

X‐ray powder diffraction (XRPD) measurements are performed at room temperature on the smallest size fraction of the sample deposited on a Bruker zero‐background sample holder using a Bruker D5005 diffractometer (Bruker Corporation, Billerica, MA, USA) with CuKα radiation, graphite monochromator, and scintillation detector. The measurements are performed from 3° to 65° with step scan mode: scan step 0.02°, counting time 10 s per step; X‐ray tube working conditions: 40 kV and 40 mA.

#### 2.2.3 FT‐IR Spectroscopy

HH powder of the smallest size is characterized by Fourier transform infrared (FT‐IR) spectroscopy, using a Nicolet (Madison, WI, USA) FT‐IR iS10 spectrometer equipped with an attenuated total reflectance (ATR) sampling accessory (Smart iTR with a diamond plate). IR spectra are obtained by c‐adding 32 scans in the range from 4000 to 650 cm^−1^ with the resolution set at 4 cm^−1^. The spectra are acquired on HH powder after automated background collection and subtraction, as included in the instrument software, without any further data pretreatment; additionally, different aliquots have been tested to check the biological variability, but no significant difference has been registered for IR peaks.

#### 2.2.4 Raman Spectroscopy

The three batches of HH powder obtained by the automated milling procedure (200, 500, and 750 µm) are characterized by Raman spectroscopy to reduce the contribution of particles dimensions in the results acquisition.

Raman spectra of the three HH powder batches were acquired using a DXR2xi Raman imaging microscope (ThermoFisher Scientific, Waltham, MA, USA) equipped with a 785 nm excitation laser operating at 30 mW and a 10× objective, with the powders deposited on silicon substrates. Each spectrum was obtained by averaging five accumulations with an acquisition time of 5 s each and applying baseline and cosmic rays correction. For each batch, Raman spectra were collected at 30 randomly selected points to assess spectral variability and sample uniformity.

While for other outputs the biological variability was not significant, Raman spectra exhibit an higher variability among different batches: for this reason, they are submitted to principal component analysis (PCA) to better evaluate powder homogeneity, particles dimensions, and milling effect on chemical structure. The input data matrix is so compiled: 30 sampling points are acquired per each of the 3 batches with different particle dimensions, leading to 90 Raman spectra in a range 3200–60 cm^−1^ with the resolution set at 1 cm^−1^. Further preprocessing prior to PCA application includes Raman shift range selection (1800–580 cm^−1^), Savitsky‐Golay filtering (polynomial degree 3, window size 15), and normalization by Standard Normal Variate (SNV), following a well‐established procedure for Raman data multivariate treatment [28, 29, 30, 31]. Software CAT has been used to run multivariate data elaboration [32].

#### 2.2.5 Thermal Analysis

Thermogravimetric analysis (TGA) is performed by a Q5000 apparatus (TA Instruments, New Castle, DE, USA) interfaced with a TA5000 data station under nitrogen flux (10 mL  min^−1^) in a platinum pan by heating about 5 mg of sample from room temperature (the working laboratory conditions) up to the total decomposition of the samples, namely, 650 °C (heating rate 10 K min^−1^ to obtain the decomposition steps separation).

HH powder of the smallest size are characterized by differential scanning calorimetry (DSC) using a Q2000 apparatus (TA Instruments, New Castle, DE, USA) interfaced with a TA5000 data station under nitrogen flux (10 mL min^−1^) in an aluminum pan by heating about 5 mg of sample from 0 °C (with the aim to have a good base line from 25 °C, the laboratory working conditions) up to 350 °C, the maximum working temperature of the instrument (heating rate 5 K  min^−1^ to separate the different evolving peaks corresponding to the different decomposition steps). TGA and DSC data were analyzed by the TA Instruments Universal Analysis software (V4.5A).

### 2.3 Composition Assessments

#### 2.3.1 CHNSO

The elemental composition (C, O, N, S) of the HHs is estimated on the finest grain sample through energy‐dispersive spectroscopy (EDX) with an X‐max 50 mm^2^ (Oxford Instruments, Oxford, UK) detector, coupled with the SEM instrument. Samples were collected from various regions of the hoof to ensure representativeness in the analysis. Results are expressed as the mean ± SD (*n* = 5). 5 spots for each sample were considered for the mean calculation.

#### 2.3.2 Protein and Lipids Content

Protein content in HH is estimated from nitrogen values obtained in the elemental analysis by applying a nitrogen factor of 6.25, as previously reported for other keratinous materials such as chicken feathers [33], sheep hair waste [34], and cattle hooves [35]. Lipid content is determined using the Bligh and Dyer method [36] on ground samples. Results, expressed as a percentage on a dry basis (db), are reported as the mean ± SD (*n* = 4). The moisture content was determined by drying the hoof samples at 105 ± 2 °C in an oven until constant weight, in accordance with the official standard method ASTM E871‐82 [37].

#### 2.3.3 Ashes

Total ash content was determined by incinerating ground HH samples (0.5–2 g) in a muffle furnace at 575 ± 25 °C until constant weight, according to the official standard method ASTM E1755‐01 for ash determination in organic materials and biomass [38]. Due to the high density and sulfur‐rich nature of the equine hoof keratin matrix, the incineration period was extended to 24 ± 6 h to ensure complete calcination.

#### 2.3.4 Higher Heating Value (HHV)

Combustion is the most widely used technology in the energy sector for converting biomass into energy. The higher heating value (HHV) represents the total amount of heat released during the complete combustion of biomass, including the cooling of combustion gases back to the initial temperature. Although HHV can be determined experimentally through calorimetry, this method is complex and time‐consuming. To simplify the process, recent approaches have developed mathematical models that estimate HHV based on the elemental composition of biomass. Elemental analysis provides a basic characterization of fuels, particularly their carbon content, which can be used in straightforward equations. The HHV of the hoof samples is estimated using the following equation as proposed by Channiwala et al. [39]:

(1) $$\begin{array}{cc} \mathrm{HHV}\;(\mathrm{MJ}/\mathrm{kg})= & 0.3491\;C+1.1783\;H+0.1005\;S\\ & \;–0.1034\;O\;–0.0151\;N \end{array}$$

where C, H, S, O, and N, respectively, refer to the carbon, hydrogen, sulfur, oxygen, and nitrogen content, expressed in ash‐free and dry basis. Regarding the hydrogen, to overcome the EDX limitation to measure the hydrogen content, a theoretical H content of 6.50% was adopted based on consolidated literature for keratins [40]. The experimental EDX data for C, S, O, and N were subsequently normalized to accommodate this hydrogen. This approach allows the use of Channiwala’s correlation, honoring the proteinaceous, sulfur‐rich matrix of the hoof samples.

#### 2.3.5 Water Sorption Kinetics

The water sorption capacity of HH samples is evaluated by monitoring their response to prolonged immersion in water, following the method of Wanger et coworkers [41] with some modifications. Upon arrival at the laboratory, hooves are brushed and cut into uniform pieces (approx. 1 cm × 0.5 cm × 0.5 cm), rinsed with distilled water, and oven‐dried at 70 °C overnight. The dried pieces are then immersed in 25 mL of distilled water at 24 °C for 10 days. Mass measurements are taken at 15‐min intervals during the first hour, followed by daily measurements. Each kinetic data point is obtained from at least five independent specimens, and immersion response is expressed as changes in specimen mass. Water sorption capacity is determined separately for bare and shod HH samples to compare their hydration behavior.

### 2.4 Metal Contamination Assessment by Microwave‐Assisted Digestion and ICP‐OES Analysis

#### 2.4.1 Analytical Procedure

Microwave‐assisted digestion of the samples is performed using a CEM Mars 5 microwave oven (CEM S.r.l., Cologno al Serio, Italy), equipped with eight 55 mL PFA‐PTFE (Perfluoroalkoxy‐Polytetrafluoroethylene) vessels (Xpress model) and an XpressVap module. Each sample is accurately weighed into PFA‐PTFE vessels; subsequently, 5 mL of HNO_3_ (67%–69% w/w, Carlo Erba Reagents S.r.l., Cornaredo, Italy) and 2 mL of H_2_O_2_ (30% w/w, Merck Life Science S.r.l., Milano, Italy) are added. The instrument operated at a maximum power output of 1600 W (100% power setting) for 15 min, reaching approximately 200 °C, as monitored by an internal infrared temperature sensor. After digestion, the resulting clear solutions are cooled, evaporated to a final volume of approximately 0.5 mL using the XpressVap module, and then diluted to 10 mL with ultrapure water in calibrated polypropylene tubes. An inductively coupled plasma optical emission spectrometer (ICP‐OES iCAP 7400, Thermo Fisher Scientific), equipped with a concentric nebulizer, cyclonic spray chamber, and ceramic duo‐torch, was used for metal measurements. Blanks and a certified reference material (BCR‐397, Trace Elements in Human Hair) are processed and analyzed alongside the samples. All results are reported on a dry weight basis (µg/g dry weight).

#### 2.4.2 HH Samples Homogeneity and Milling Contamination Assessment

HH samples homogeneity in terms of metal content is assessed by quantifying the major metal contaminants (Mn, Cu, Zn, Al, and Fe) in three different parts of the same shod hoof. More in detail, four aliquots are sampled from the wall in the facial region of the hoof, while four others in the lateral region from both right and left sides. Additionally, two aliquots per each region are manually milled before digestion to evaluate possible milling contamination, in this case using WF. The so‐acquired 8 samples are submitted to the general procedure described in Section 2.5.1.

The calculated metal contents, expressed as μg/g, are first independently evaluated and then submitted to PCA, after autoscaling, to detect similarities or dissimilarities between different sampling regions and correlations among metal ions. Software CAT has been used to run multivariate data elaboration [32].

#### 2.4.3 Metal Content Determination in HH Samples with Various Shoeing

HH samples are collected from 16 different horses (facial region) to include in this first panel test bare horses (4 samples), horses with traditional iron‐made shoes (4 samples), and with innovative ones, mainly made of aluminum, copper, plastic, or including the use of leather between the hoof and the shoe (8 samples). The microwave‐assisted digestion and ICP‐OES analysis (see Section 2.4.1) are performed on HH small pieces after cleaning, avoiding milling. For the so‐acquired samples, both major (Mn, Cu, Zn, Al, and Fe) and minor (Cd, Pb, Cr, and Ni) metal contaminants are quantified to preliminarily evaluate possible correlation between their content and the various shoeing practices.

Also, in this case, after a first evaluation, the determined metal contents are autoscaled and submitted to PCA to better extract information about the target correlation.

## 3 Results and Discussion

### 3.1 HH Collection, Pretreatment and Milling

**FIGURE 1:**
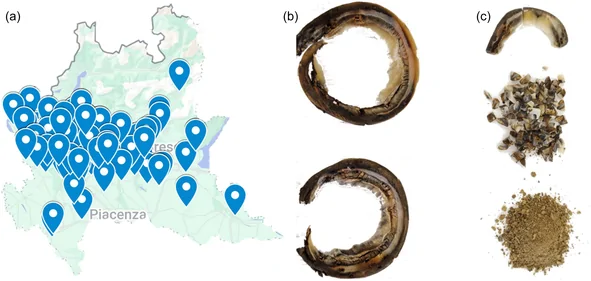
(a) Tentative representation of stables, riding centers and horse breedings in Lombardia; visual examples of HH waste as obtained directly from the trimming operations (b) and comparison between HH waste entire samples, small pieaces and powder (c).

The investigation on HH is performed sampling this peculiar waste material from stables located in the Lombardia region (Italy): this region served as the sample source both for logistic reasons and for the widespread diffusion of stables, horse breeding, and riding centers in this geographic area, as depicted in Figure 1a. Per each hoof, the samples present an average weight of around 50 g and a hoof‐shaped form, as depicted in Figure 1b. To facilitate handling and storage, the samples are cut into smaller pieces of around 200 mg each and eventually milled to get a heterogeneous powder, as summarized in Figure 1c. This top‐down, or mechanical approach, as already discussed by Al Faruque and coauthors, is easier, safer, quicker, more environmentally friendly, and allows to retain original material inherent characteristics [19].

**FIGURE 2:**
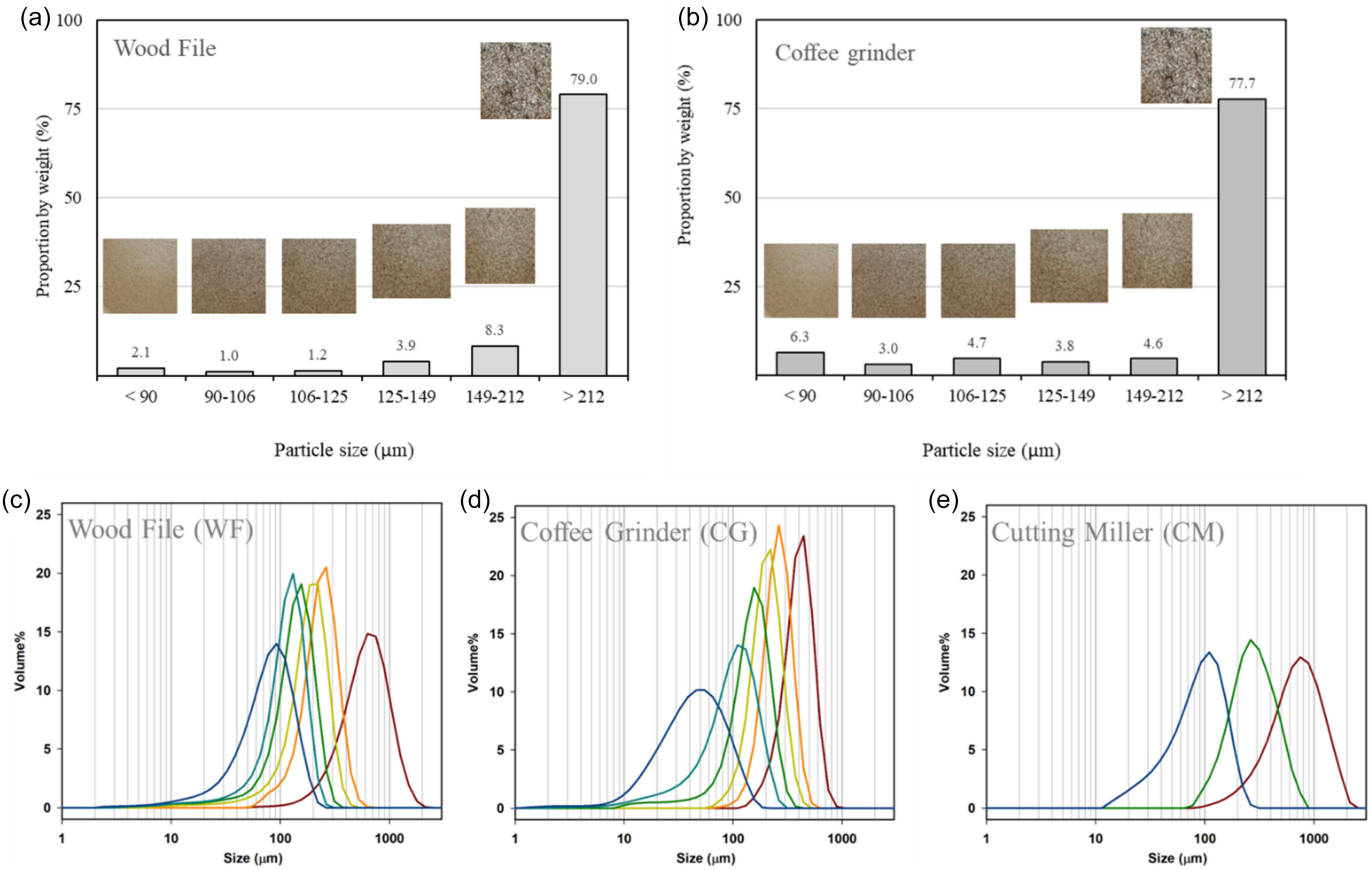
PSD characterization by a series of bottom sieves (a,b) or by laser diffraction analysis (c‐e) for HH powder obtained using WF (a,c) CG (b,d) and automated cutting millers (e).

The peculiar hardness of HH waste makes milling procedure a critical task: in this investigation, we explore both manual milling by means of a WF and automatic grinding by CG, while bowl milling was not successful. The so‐obtained powders are first characterized using a series of bottom sieves (Figure 2a,b): in both cases, more than 75% of the powder present a size above 212 μm, while the smallest fraction, below 90 μm, corresponds to far less than 10% of the total powder. These results suggest that both WF and CG cannot produce a small and homogeneous powder, for which a rotatory cutting miller is required [19, 20], while they can be suitable for a preliminary milling operation.

A deeper characterization of PSD is achieved using laser diffraction analysis, as presented in Figure 2c,d, for WF and CG milling, respectively. The higher sensitivity of this technique allows to distinguish between the two powders, with the second one characterized by a lower PSD. Indeed, the D50 value for the overall sample obtained with the CG is 277.3 μm against 360.9 μm for that produced by WF. More in detail, the D50 of the smallest fraction is 40.3 μm for CG versus 72.6 μm for WF, while the same value for the biggest fraction is 370.5 μm for CG and 570.2 μm for WF. These results suggest that CG is preferable to obtain a powder with smaller particles, even if still inhomogeneous. The so‐obtained powder presents around 4‐fold bigger dimensions than those reported in the first pulverization of alpaca fibers using rotatory cutting miller [19] and almost 5‐fold bigger than steamed sheep hooves powder [22] but still acceptable considering the much harder type of keratinous waste and the softer milling strategy.

For a more reliable comparison between different keratinous wastes, the PSD results for milled HH waste are shown in Figure 2e: even using an automated milling procedure, each fraction presents a heterogeneous distribution with a D50 of 649.4, 253.9, and 83.2 μm, respectively. These results suggest that, even with an automated pipeline, particles dimensions are not significantly decreased, compared to the manual approach, and remain much bigger than literature examples in which the same millings were used but on different keratinous wastes, confirming the extreme hardness of the material [19, 22].

These results are particularly interesting if considered with an eye to HH upcycling: manual procedures can be successfully applied in a lab‐scale environment, while an automated pipeline is mandatory to scale the production, even if the overall PSD is not particularly affected by the milling approach and obtaining a satisfying amount of particles below 100 μm still represents a critical task.

### 3.2 HHs Physicochemical Characterization

**FIGURE 3:**
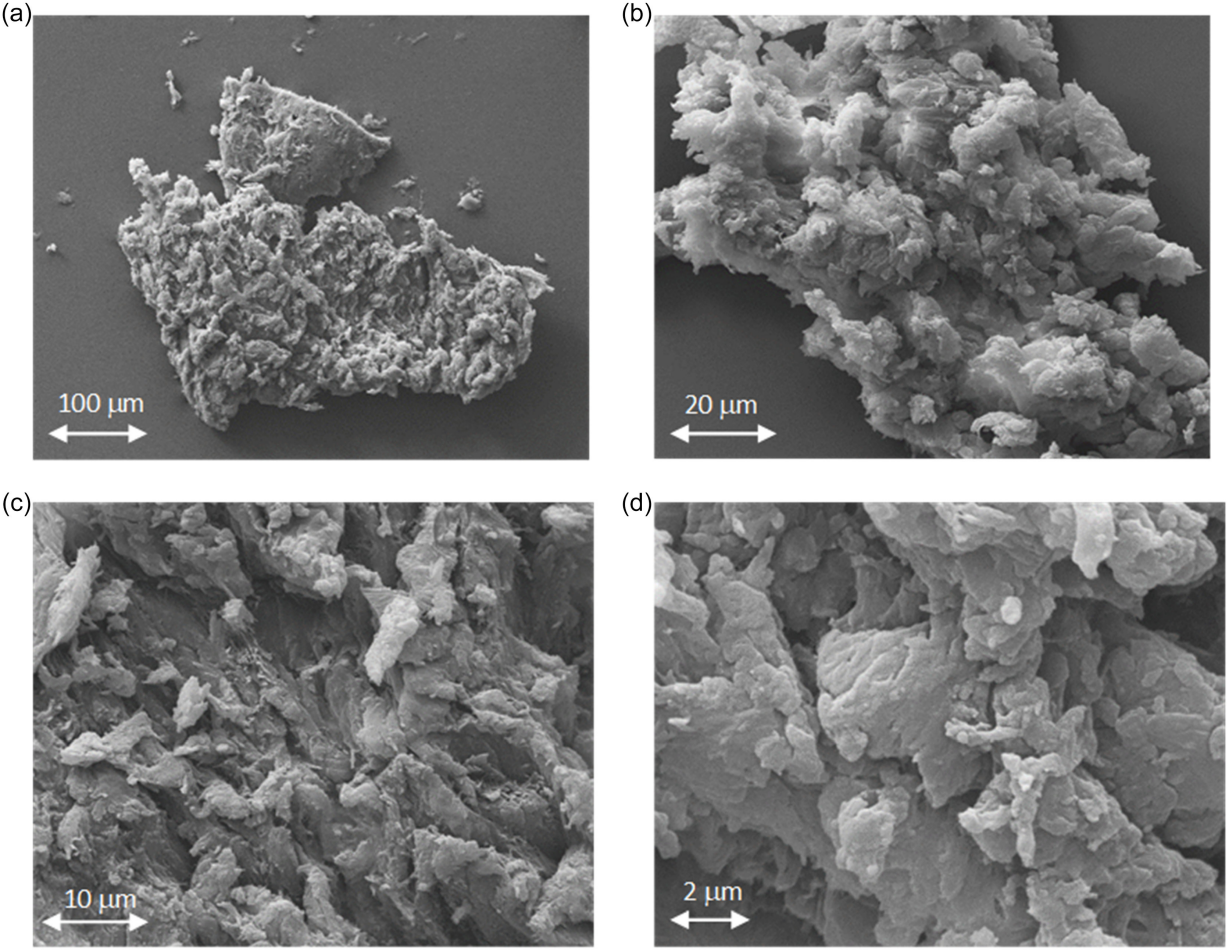
SEM images of the HH sample (larger grain size) at 500× (a), 2500× (b), 5000× (c), and 15000× (d).

HH samples have been characterized by SEM coupled with EDX to jointly evaluate the morphology of the material and its elemental composition, as proposed in the literature for similar materials [3, 19]. The SEM images (Figure 3) of the waste after the raw milling (strategy a) show the presence of agglomerates from 100 to 400 µm long and up to 200 µm large (a). Their surface appears composed of interconnected plaques maximum 5 µm long (d), covered by small prismatic or rounded grains (b), often interconnected to form chains or cylinders long up to 10 µm.

**FIGURE 4:**
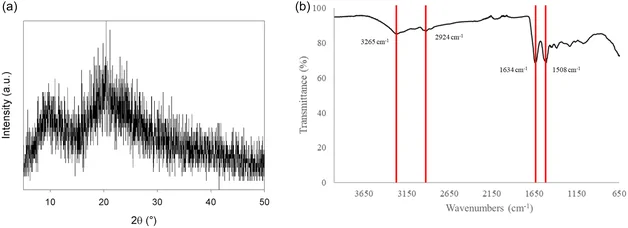
XRPD patterns (a) and FT‐IR spectrum (b) for the HH sample.

XRPD analysis on the HH sample shows its semicrystalline character, with a band centerd at 9° attributable to the alpha‐helix structure of keratin and a huge band centerd at 21° composed of the overlapping of the signals of the alpha‐helix structure and the beta‐sheet structure of the biopolymer, as described in literature [23, 42, 43] and observed for other materials rich in keratin like feathers biomass from the poultry industry [44] and wool [45], as shown in Figure 4a.

Since no significant differences were detected between different characterized samples, the general FT‐IR spectrum of HH is shown in Figure 4b: the registered profile perfectly agrees with other examples of keratin‐based materials reported in the literature [3, 14, 16, 19, 20]. According to previous investigations [3, 19, 46], the bands at 3265 and 2924 cm^−1^ represent Amide A and B signals and can be attributable to N─H bond stretching. The two bands at 1634 and 1508 cm^−1^ can correspond to Amide I and II, referring to the stretching of the C═O and C─N groups, respectively. Amides III–VII at lower wavelengths cannot be clearly distinguished in the raw materials. Nevertheless, the similarity between milled HH and pure keratin [20, 46] IR spectroscopy confirms that keratin represents the main component of this waste material, while this technique is not sensitive enough to distinguish other minor components.

**FIGURE 5:**
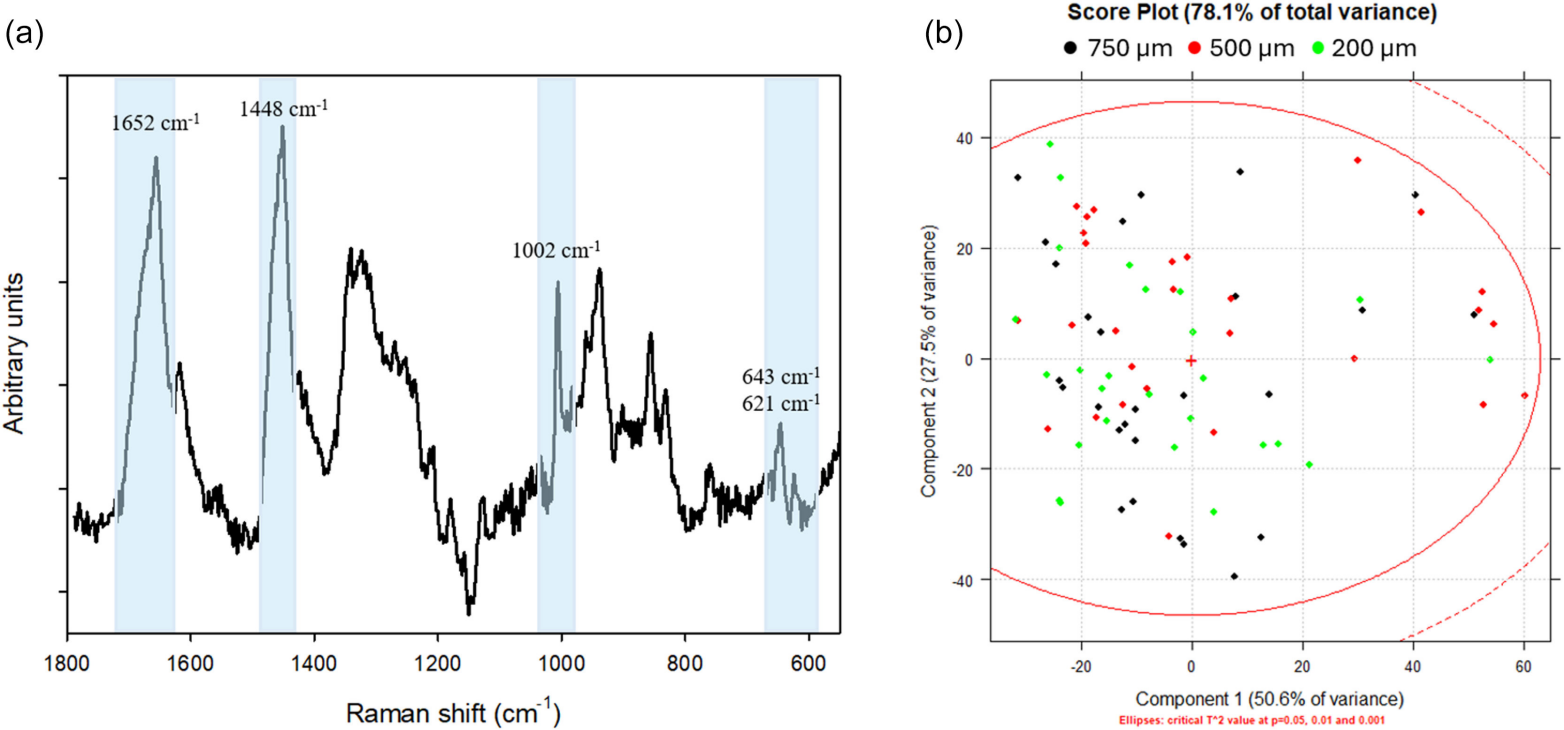
Representative Raman spectrum (a) and PCA score plot on the first two components (b) run on Raman spectra for the HH sample.

The representative Raman spectrum of HH, shown in Figure 5a, further confirms the keratinous nature of HH waste: according to previous investigations [47, 48], the signal at 1652 cm^−1^ can be attributed to C═O stretching vibration of Amide I, the band at 1448 cm^−1^ refers to CH_2_ scissoring vibration of aliphatic amino acid side chains while the peak at 1002 cm^−1^ is generally considered a protein marker, being attribute to aromatic C–C stretching mode of phenylalanine. Lastly, the presence of sulfur atoms is confirmed by the presence of C–S stretching vibrations at 643 and 621 cm^−1^ [47, 48].

Besides confirming the keratinous nature of HH waste and the absence of any other significant components, Raman spectra are exploited to characterize the effect of automated milling on HH powder’s chemical structure and batch homogeneity. The score plot on the first two components for PCA on sampling points, explaining 78.1% variance, clearly shows a complete overlapp of spectral components recorded within randomly selected regions of interest across different fractions and the absence of T^2^ outliers. This result leads to two main findings: (i) harsh milling procedures required to reduce particle dimensions do not affect HH chemical structure and (ii) HH powder chemical structure can be considered reasonably homogeneous at a confidence level of 95%.

**FIGURE 6:**
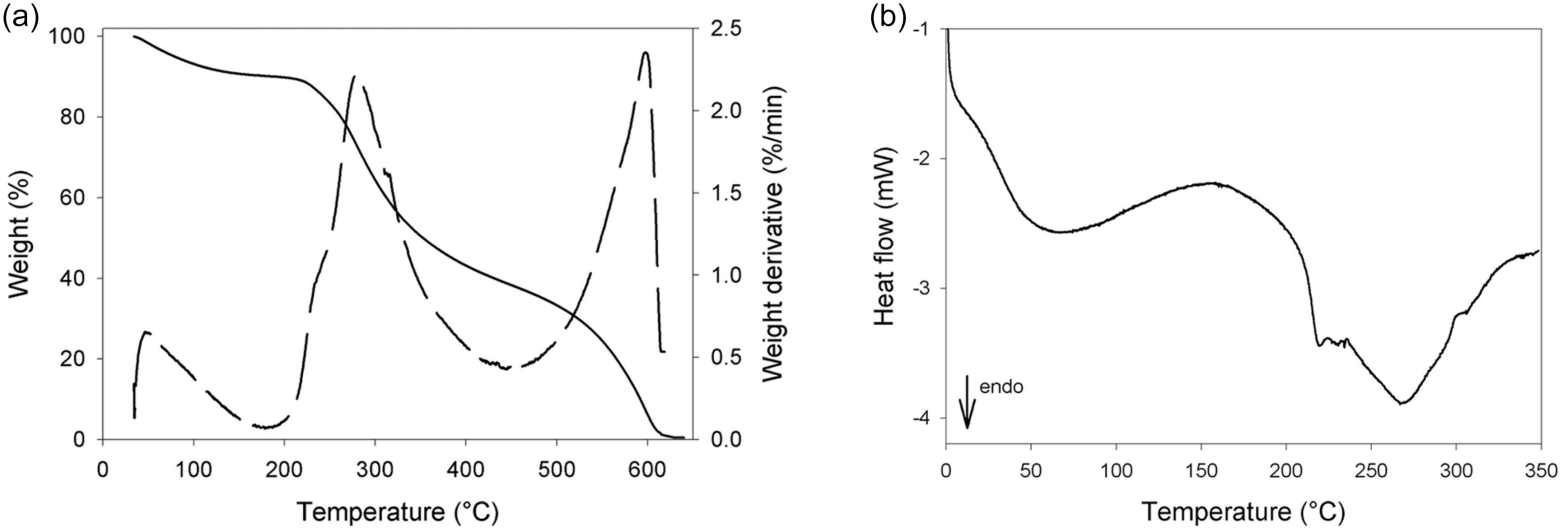
TGA (a) full line: thermogravimetric curve; dotted line: derivative of the weight loss with respect to time DTG) and DSC profiles (b) for the HH sample.

The thermogravimetric curve in Figure 6a (full line) shows an initial mass loss of around 10% from room temperature up to 175 °C attributable to the release of adsorbed and bound water [49], followed by the full decomposition of keratin [50] in two steps, the first one (50% of loss) from 180 to 450 °C, with the evolution of more subsequent reactions like the breakage of disulfide bond and β‐sheet conformation [42, 51], as evident in the DTG trace (dotted line), and the second one from 450 to 630 °C (40% loss) due to the biopolymer skeletal degradation [52]. This trend is in agreement with the reports for keratin from bovine hooves [49] and from waste broiler chicken feathers [53], even if for those materials the two stages of decomposition are shifted at higher temperatures.

The calorimetric analyses (DSC) in Figure 6b are in agreement with the TGA data, showing a first huge endothermic peak (from 12.50 to 150 °C, centerd at 56.70 °C) attributable to the water release and a complex endothermic signal, due to the evolution of different decomposition processes, from 155 °C to the end of the analysis.

### 3.3 Horse Hooves Composition Assessment

As expected for keratin‐rich materials [34], the EDX analysis reveals that the HH samples are composed mainly of C (52.94 ± 2.44 wt%; 60.49 ± 1.88 at%) and O (34.13 ± 1.62 wt%; 29.27 ± 1.92 at%), with high content of N (8.56 ± 3.16 wt%; 8.40 ± 2.88 at%) and a lower amount of S (3.96 ± 0.24 wt%; 1.70 ± 0.14 at%) ascribed to the presence of cysteine. In our samples, traces of K (0.39 ± 0.07 wt% and 0.14 ± 0.02 at%) are also present. More precise and reliable results can be obtained using an elemental analyzer, but considering the preliminary nature of the investigation, we considered this assessment an acceptable compromise.

As already hinted in the literature [54], when compared with other keratinous materials, the HH samples exhibit a markedly higher carbon content (33%–36% increase), accompanied by a reduction in nitrogen (33%–41%) and oxygen (15%–20%) relative to cow hooves [55] and keratin powder from sheep hooves [22]. Nevertheless, this comparison should be evaluated as qualitative since different methodologies are used in the literature to assess the elemental composition.

The elevated carbon concentration contributes to a HHV of 21.9 MJ/kg. Although no prior data are available on the HHV of HHs, the value obtained in this study exceeded those reported for other keratinous materials such as cow hooves (19.51 MJ/kg) [55] and duck feathers (14.87–16.24 MJ/kg) [14]. Moreover, HH displays higher HHV compared to other residues, including tobacco leaf (15.00 MJ/kg), rice husk bran (15.29 MJ/kg), and forest residues (19.50 MJ/kg), as well as to several bio‐based fuels and materials such as coal (14.77 MJ/kg), rapeseed (16.61 MJ/kg), miscanthus pellets (19.00 MJ/kg), and oak wood (19.17‍–‍19.24 MJ/kg) [56, 57]. This comparatively high HHV highlights the strong potential of HHs as a renewable, cost‐effective biomass feedstock for energy production.

The moisture content of as‐received HH samples is 25.7% ± 2.8%, with no significant differences between shod (25.7% ± 2.7%) and barefoot (25.6% ± 3.0%) pieces (*p* < 0.05). Similar values were reported by Spörndly‐Nees et al. [54] in hoof wall samples from frequently (27.0%) and occasionally (27.7%) barefoot‐racing horses, with no significant differences observed between groups. The residual moisture in the dried HH samples used for characterization is below 5% (1.0% ± 3.0%).

Regarding chemical composition, proteins are the major component, representing 56% ± 4% (db), while lipids are present at less than 2% (1.1% ± 0.2%). The protein content is lower than that reported by Tocci and Sargentini [58] for HH from four horse breeds (98.24%–98.86%, db), where no lipids are detected and ash content is minimal. However, our findings are consistent with Yilma et al. [35], who reported lower protein content (75.05%) and low fat (1.5%) in cattle hooves, as well as with Spörndly‐Nees et al. [54], who observed fat levels ranging from 0.7% to 1.1% in HH samples.

Total ash content averages 6% ± 5%, showing substantial variability across samples. Separate analysis of shod and barefoot HH reveals significant differences (*p* < 0.05), with shod samples containing 3.0% ± 1.1% ash and barefoot samples 12% ± 2%. Similar variability has been reported for ash content in other animal hooves, including cow (2.24%) [55], donkey (1.2%) [59], and cattle (6.554%) [35]. Rueda‐Carrillo et al. [26] further demonstrated that mineral content in HH can vary significantly depending on sampling region (Ca, Se, Zn, K, and Na), riding discipline (Mg), age (Na), and sex (Zn), while no significant breed differences are found. Moreover, environmental factors such as soil mineral composition, pasture conditions, and rainfall at the time of sampling may also influence mineral uptake. These factors likely explain the higher ash content in barefoot samples compared to shod ones, where additional metal contamination from horseshoes can be expected. A more detailed discussion of the metal profile and differences between shod and barefoot samples will be presented in the following sections.

### 3.4 HHs Water Sorption Capacity

**FIGURE 7:**
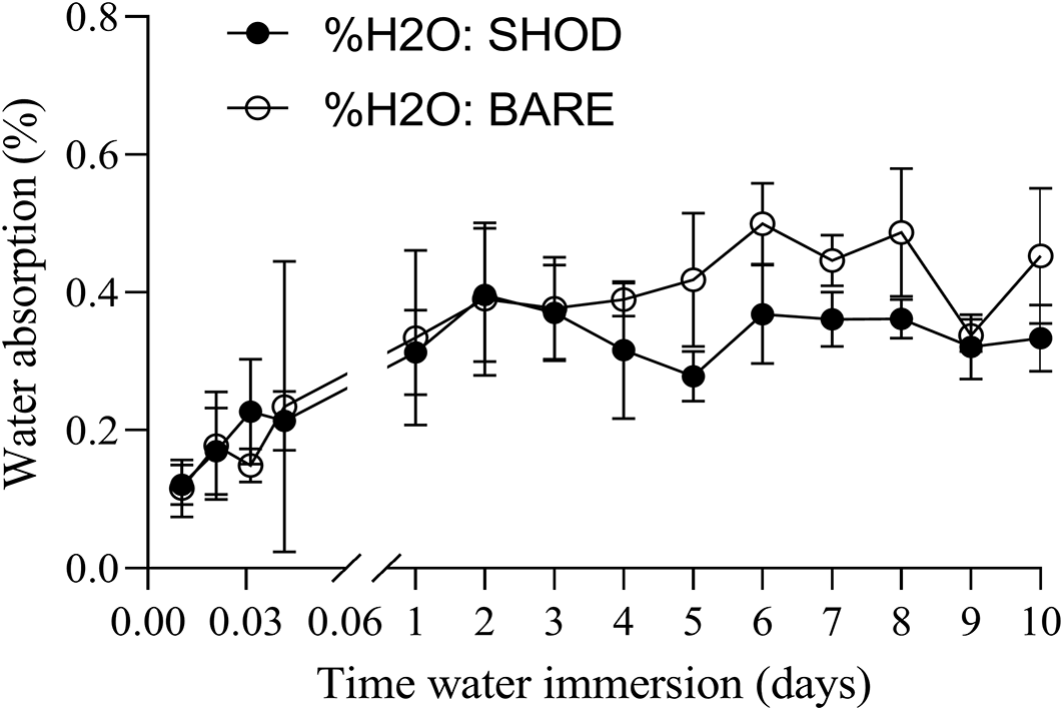
Water sorption kinetics during the immersion period of hooves from shod (•) and barefoot (○) horses. Within each group, differences are indicated by different letters (lowercase for shod, uppercase for barefoot; *p* < 0.05). Comparisons between shod and barefoot samples are marked as significant at *p* < 0.05 (*) and *p* < 0.01 (**). Data are presented as mean ± SD (*n* ≥ 5).

The water sorption kinetics during the immersion period for both shod and barefoot HH samples are shown in Figure 7. Overall, both groups followed a similar trend up to 3 days of immersion. Significant differences (*p* < 0.05) appear from day 4 onwards, with barefoot hooves exhibiting 8.6%–14.0% higher water sorption capacity than shod hooves (except on day 9). In barefoot samples, water sorption continues to increase slightly beyond day 4, reaching a maximum of 49.96% on day 6. In contrast, shod samples reach their maximum (39.59%) on day 2, after which the values remain stable or show a slight decline. ANOVA analysis confirms that both sample type and immersion time have significant effects (*p* < 0.01) on water sorption, whereas their interaction is not statistically significant. The observed variability may be related to differences in environmental exposure before sampling since hooves from horses in wet environments absorb less water than those from arid regions [41].

Hydration status influences hoof properties by modifying elasticity and resistance, factors that must be considered when evaluating their use as a material to optimize performance. A similar trend was reported by Wagner and Hood [41], who described a rapid initial absorption phase with no significant increase in sorption beyond the first immersion day. Once the hoof becomes saturated, a plateau is reached, occasionally followed by minor, nonsignificant decreases, consistent with the behavior observed in shod samples in this study. These results highlight the capacity of HHs, from both shod and barefoot horses, to absorb water, a property that may determine their potential applications as biomaterials.

### 3.5 Metal Contamination in HH Samples

**FIGURE 8:**
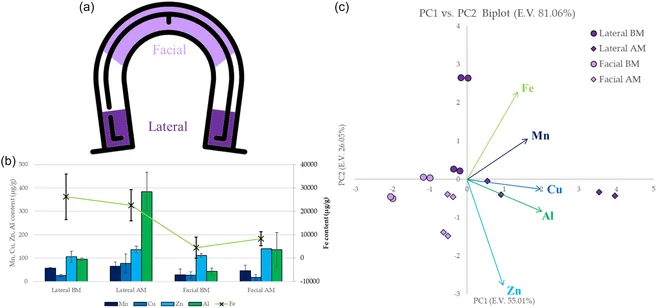
(a) Sampling approach for metal content homogeneity assessment in shod HH; (b) Average metal contents in different HH regions (lateral and facial) before (BM) and after milling (AM); (c) PCA biplot on the first two components for metal content homogeneity and milling‐derived contamination.

HH homogeneity and milling‐derived contamination, in terms of metal content, are first assessed by analyzing aliquots in different hoof regions, namely, lateral and facial, before (BM) and after milling (AM); the sampling procedure is summarized in Figure 8a. The bar plot in Figure 8b allows a glaring visualization of the effects related to sampling area and milling‐derived contamination: lateral regions are generally richer in metals rather than the facial area, especially for iron, and milling procedure increases Cu, Zn, and Al content.

The application of PCA on the same dataset allows to further highlight the correlation between metal content and sampling area or milling, as shown in the PC1 versus PC2 biplot in Figure 8c: the main distinction between the lateral (light violet) and facial area (violet) is mainly attributable to Fe and Mn content, while Cu and Al have a slighter impact. Such a glaring difference is never reported in the literature since most of the investigations focus on sampling always the same hoof region [26, 58, 60, 61, 62, 63]. Interestingly, the separation between samples before (circles) and after milling (diamonds) is mainly associated with Cu, Al, and Zn content.

**FIGURE 9:**
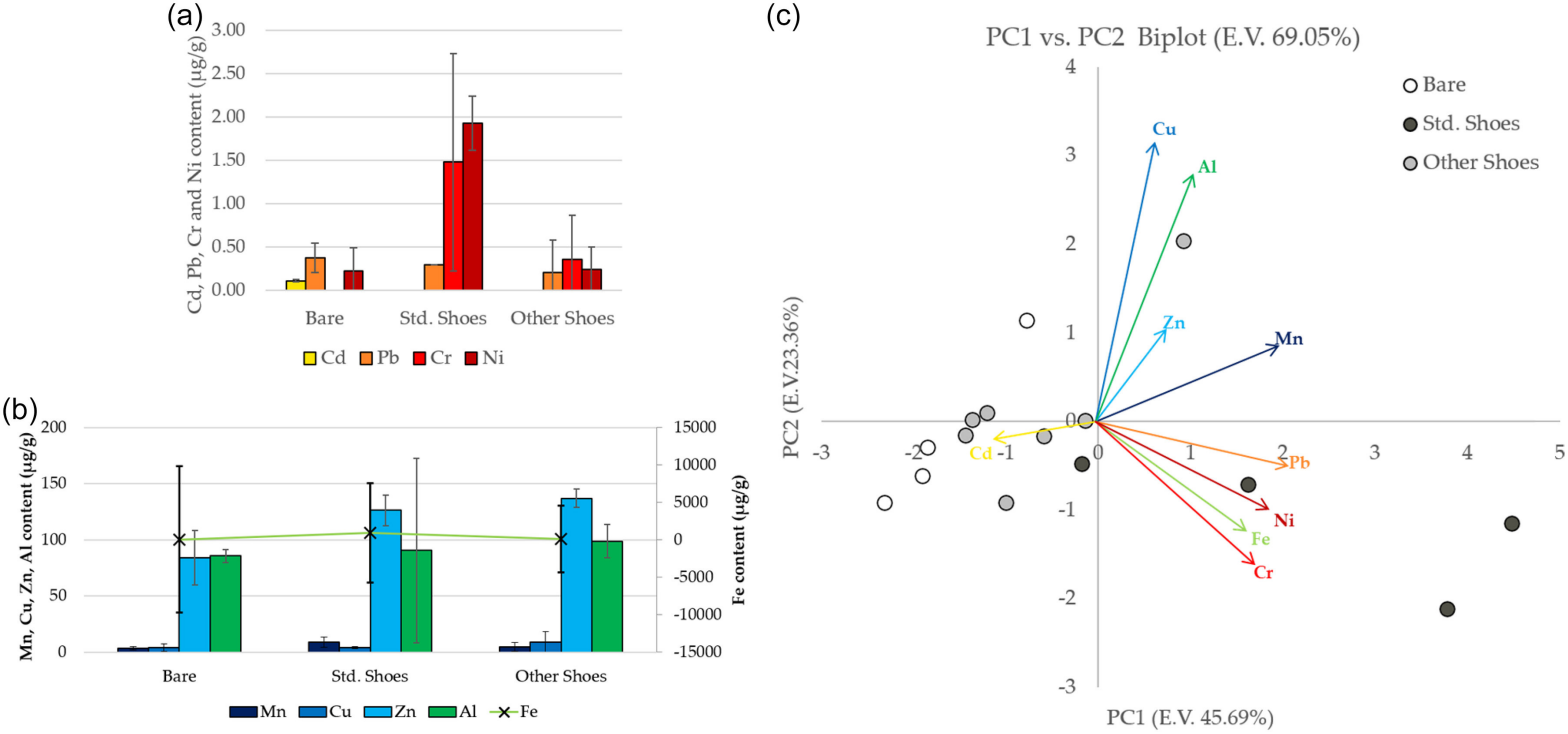
Average contents for minor (a) and major (b) metal contaminants in HH with various shoeing practices and (c) PCA biplot on the first two components for shoeing‐dependant metal contamination assessment.

The set of analyses reveals that HH samples are highly inhomogeneous, and the milling procedure results in significant contamination: keeping these bullet points in our minds, the comparison between hooves from horses with different shoeing is performed, always sampling the facial region and avoiding milling. Metal contents are summarized in the bar plots in Figure 9a,b, for minor and major contaminants, respectively. Several conclusions can be drawn from these data:

1. Cd is detected only in bare horses.
2. Pb, Mn, and Cu content seems to be unaffected by shoeing.
3. Cr and Ni are much more concentrated in horses with traditional shoes.
4. Zn content is higher in shod horses, regardless of the type of shoe.
5. The content of Fe in all samples and Al in traditionally shod horses, is highly variable.

Also in this case, the application of PCA allows an easier visualization of HH features, as depicted in the PC1 versus PC2 biplot in Figure 9c: PC1 clearly represents metal contamination, mainly attributable to Cr, Fe, Ni, Pb, and Mn and less dependent from Zn, Al, and Cu, except for Cd, that shows an opposite trend. Alongside this direction, bare HH are obviously the less contaminated, regularly shod HH the more, and HH with innovative shoes are located in between these two groups. PC2 instead better describes the separation between Cu, Al, Zn, and Mn versus the other metals and allows an even clearer separation of traditionally shod horses.

Despite the preliminary nature of these data, the results seem to be consistent, especially for contamination from milling and traditional shoes; unfortunately, a more detailed comparison with literature cannot be performed since previous investigations focus their attention on different metals limiting the analysis to bare horses [24, 25, 26] and after milling [26, 54, 58, 62, 63, 64]. Nevertheless, the data acquired confirmed that metal contamination is an issue that must be addressed when proposing upcycling strategies for this type of material, not only in the case of shod HH but also for the bare ones. This means that HH waste does not represent the most straightforward candidate for applications in which purification is required, such as in food‐related, biomedical, and cosmetic sectors [12, 13], and further investigations are mandatory for optimizing cleaning procedures to remove these contaminants before HH upcycling.

## 4 Conclusions

This work provides the first multitechnique physicochemical, structural, and thermal characterization of HH waste as a keratin‐rich material. The main findings can be summarized as follows:

1. Milling: HH hardness hampers fine powder production, requiring advanced milling equipment for homogeneous particle sizes, though coarse milling is sufficient for keratin extraction.
2. Structure and composition: SEM‐EDX, XRPD, Raman, and FT‐IR analyses confirmed the keratinous nature of HH, with high carbon content and moderate protein levels. The high HHV (21.9 MJ/kg) suggests a strong potential for energy recovery.
3. Hydrophilicity: Water sorption capacity differed between barefoot and shod hooves, with barefoot ones displaying higher hydration.
4. Metal contamination: Considerable variability was found across sampling regions and shoeing conditions, with barefoot hooves generally less contaminated. Milling procedures themselves may introduce additional metal traces.

Overall, HHs represent a sustainable and cruelty‐free keratin source with promising prospects in energy conversion and material science. However, variability in composition and metal contamination remain critical limitations, requiring further optimization of collection and pretreatment before reliable upcycling strategies can be implemented.

## Author Contributions

Conceptualization: Esther Trigueros and Lisa Rita Magnaghi. Methodology: Esther Trigueros, Lorenzo De Vita, Lavinia Rita Doveri, Michela Sturini, and Chiara Milanese. Formal analysis: Esther Trigueros, Lorenzo De Vita, Lavinia Rita Doveri, Michela Sturini, and Chiara Milanese. Investigation: Esther Trigueros, Lisa Rita Magnaghi, and Chiara Milanese. Resources: Michela Sturini, Chiara Milanese, and Raffaela Biesuz. Data curation: Esther Trigueros, Lisa Rita Magnaghi, Lorenzo De Vita, Lavinia Rita Doveri, and Chiara Milanese. Writing – original draft: Esther Trigueros, Lisa Rita Magnaghi, and Chiara Milanese. Writing – review and editing: Lorenzo De Vita, Lavinia Rita Doveri, Michela Sturini, and Raffaela Biesuz. Supervision: Raffaela Biesuz.

## Conflicts of interest

The authors declare no conflicts of interest.

## Data Availability

The data that support the findings of this study are available from the corresponding author upon reasonable request.
